# Tree diversity promotes insect herbivory in subtropical forests of south-east China

**DOI:** 10.1111/j.1365-2745.2010.01659.x

**Published:** 2010-07

**Authors:** Andreas Schuldt, Martin Baruffol, Martin Böhnke, Helge Bruelheide, Werner Härdtle, Anne C Lang, Karin Nadrowski, Goddert von Oheimb, Winfried Voigt, Hongzhang Zhou, Thorsten Assmann, Jason Fridley

**Affiliations:** 1 Leuphana University Lüneburg, Institute of Ecology and Environmental Chemistry, Scharnhorststr1, D-21335 Lüneburg, Germany; 2 University of Zurich, Institute of Environmental Sciences, Winterthuererstr190, CH-8057 Zurich, Switzerland; 3 University of Halle, Institute of Biology/Geobotany and Botanical GardenAm Kirchtor 1, D-06108 Halle, Germany; 4 Max-Planck-Institute for BiogeochemistryPB 100641, D-07740 Jena, Germany; 5 University of Jena, Institute of EcologyDornburger Str. 159, D-07749 Jena, Germany; 6 Chinese Academy of Sciences, Institute of ZoologyBeijing 100101, China

**Keywords:** BEF China, biodiversity, ecosystem functioning, Gutianshan, resource concentration, succession, trophic interactions, Zhejiang

## Abstract

**1.**Insect herbivory can strongly affect ecosystem processes, and its relationship with plant diversity is a central topic in biodiversity–functioning research. However, very little is known about this relationship from complex ecosystems dominated by long-lived individuals, such as forests, especially over gradients of high plant diversity.

**2.**We analysed insect herbivory on saplings of 10 tree and shrub species across 27 forest stands differing in age and tree species richness in an extraordinarily diverse subtropical forest ecosystem in China. We tested whether plant species richness significantly influences folivory in these highly diverse forests or whether other factors play a more important role at such high levels of phytodiversity.

**3.**Leaf damage was assessed on 58 297 leaves of 1284 saplings at the end of the rainy season in 2008, together with structural and abiotic stand characteristics.

**4.**Species-specific mean damage of leaf area ranged from 3% to 16%. Herbivory increased with plant species richness even after accounting for potentially confounding effects of stand characteristics, of which stand age-related aspects most clearly covaried with herbivory. Intraspecific density dependence or other abiotic factors did not significantly influence overall herbivory across forest stands.

**5.***Synthesis*.The positive herbivory–plant diversity relationship indicates that effects related to hypotheses of resource concentration, according to which a reduction in damage by specialized herbivores might be expected as host plant concentration decreases with increasing plant diversity, do not seem to be major determinants for overall herbivory levels in our phytodiverse subtropical forest ecosystem. We discuss the potential role of host specificity of dominant herbivores, which are often expected to show a high degree of specialization in many (sub)tropical forests. In the forest system we studied, a much higher impact of polyphagous species than traditionally assumed might explain the observed patterns, as these species can profit from a broad dietary mix provided by high plant diversity. Further testing is needed to experimentally verify this assumption.

## Introduction

Increasing awareness that the human-induced loss of biodiversity may affect important ecosystem services has triggered extensive research on the relationship between biodiversity and ecosystem functioning. Focusing primarily on the producer level, much progress has been made in understanding the effects of plant diversity on productivity and nutrient cycling (Hooper *et al.* 2005; Hector *et al.* 2007). However, ecosystem processes such as plant production or nutrient cycling are strongly influenced by complex interactions between trophic levels, which need to be considered adequately to fully understand diversity–functioning relationships (Thebault & Loreau 2006; Duffy *et al.* 2007). Invertebrates, representing the bulk of faunal diversity, play a major role in this respect (Weisser & Siemann 2004). An interaction of considerable importance is herbivory by phytophagous insects. This can have a profound impact on ecosystem processes, especially as herbivores may directly influence growth and species composition at the producer level (Coley & Barone 1996; Mulder *et al.* 1999; Hartley & Jones 2004; Frost & Hunter 2008).

Relationships between plant diversity and herbivores have been studied predominantly in agricultural and grassland systems (Andow 1991; Tonhasca & Byrne 1994; Scherber *et al.* 2006b; Unsicker *et al.* 2006). Many of these studies found a decrease in herbivores and herbivory with increasing plant species richness (e.g. Andow 1991; Hambäck, Agren & Ericson 2000; Unsicker *et al.* 2006) and often related this to resource–concentration effects as suggested by Root’s (1973) hypothesis for specialized herbivores. With increasing plant diversity this hypothesis predicts a decrease in specialist herbivore loads (which can result in reduced herbivory), as host finding can be hindered by the increasing number of non-host plants (Root 1973). In contrast, other studies (often those incorporating much more diverse plots than usual intercropping experiments) report the opposite effect of increasing herbivore loads or damage with increasing plant diversity (e.g. Mulder *et al.* 1999; Otway, Hector & Lawton 2005; Scherber *et al.* 2006b). Colonization and population dynamics might explain such patterns in specialist herbivores (Otway, Hector & Lawton 2005), whereas generalist herbivores can directly profit from dietary mixing and increase their consumption in more diverse plots (Unsicker *et al.* 2008) or spill over from more to less preferred plant species (White & Whitham 2000).

Recently, forests have come into the focus of the diversity–functioning debate as more complex systems dominated by long-lived individuals and providing crucial ecosystem services (Scherer-Lorenzen, Körner & Schulze 2005). Varying effects of plant diversity on herbivore loads or damage have also been reported for these systems (Jactel & Brockerhoff 2007; Vehviläinen, Koricheva & Ruohomaki 2007; Sobek *et al.* 2009). However, analyses of forest diversity have so far mostly considered only low levels of plant diversity, often restricted to comparisons between monocultures and two- or three-species mixtures (reviewed by Jactel, Brockerhoff & Duelli 2005). In contrast to grassland systems, there is a lack of studies on plant–insect interactions over gradients of high tree diversity. Yet, this issue is of high concern for the extraordinarily species-rich subtropical and tropical forests, where herbivory is one of the dominant interactions (Coley & Barone 1996; Eichhorn, Compton & Hartley 2006). A wealth of studies in these species-rich forests have analysed effects of distance and density dependence on the growth and survival of young trees neighbouring conspecific adults (cf. Hyatt *et al.* 2003). However, differences in stand diversity have not been considered in these studies. Although not generally confirmed as a community-wide effect (Hyatt *et al.* 2003), several studies found higher herbivory and mortality of saplings closer to adult trees of the same species (e.g. Blundell & Peart 2004; Norghauer, Malcolm & Zimmerman 2006), concordant with the hypothesis of Janzen (1970) and Connell (1971) predicting herbivore spill over from conspecific trees to neighbouring saplings. This is also concordant with resource–concentration theory, as specialized herbivores are often considered to dominate herbivore communities in these forests (Barone 1998, 2000; Dyer *et al.* 2007). These studies imply that there might also be an effect of tree diversity on herbivory at medium to high species richness of trees, as the abundance of single tree species is likely to decrease in forest stands with increasing tree diversity. Interestingly, to our knowledge, this issue has not been addressed directly for highly diverse subtropical forests.

Here, we analyse herbivory on saplings of 10 tree and shrub species in three successional stages of semi-natural forest in subtropical China along a tree richness gradient from medium to high diversity (25–68 woody species per 900 m²). We chose saplings because of their importance in maintaining high stand diversity and because they represent an especially vulnerable stage within the tree life cycle. We tested whether potential effects of tree species richness on insect herbivory are detectable even in such extraordinarily species-rich subtropical forests or whether only structural (e.g. stand density) and abiotic factors play an important role for herbivory at such high levels of phytodiversity. Strong effects of plant diversity on herbivory have especially been reported from studies incorporating monocultures or low-diversity treatments. However, whether these effects persist or level out in highly diverse plant communities (cf. Hooper *et al.* 2005) has not been studied sufficiently (Unsicker *et al.* 2006). Our study across a gradient of medium to high tree diversity provides insight into the herbivory–plant diversity relationship beyond the level approached in most previous studies, but which is very relevant for the phytodiverse (sub)tropical forests. In addition to richness effects, intraspecific density-dependent effects of the proportion of the target species in the tree and shrub layer on herbivore damage of saplings might emerge. We assessed the relative statistical support for the hypotheses that (i) both plot conditions and species richness or target species density, or (ii) plot conditions alone, or (iii) only richness and/or target species density alone are important predictors of the observed pattern in herbivory across the study plots. Considering the reported high specialization of many insect herbivores in similarly species-rich forests, we might expect a decrease in overall herbivore damage on saplings with increasing plant species richness across our diversity gradient. To our knowledge, our study is the first to test explicitly the effects of tree diversity on this important plant–insect interaction for a very species-rich subtropical forest ecosystem.

## Materials and methods

### Study site and plot selection

Our study was conducted in the Gutianshan National Nature Reserve (29°14′ N, 118°07′ E), Zhejiang Province, in south-east China. The reserve, established in 1975 as a National Forest Reserve, comprises about 8000 ha of semi-evergreen broad-leaved forest at an elevation of 300–1260 m a.s.l. It is characterized by subtropical monsoon climate, with a mean annual temperature of 15.3 °C and mean annual precipitation of about 2000 mm. The parent rock of the mountain range is granite, with soil pH ranging from 5.5 to 6.5 (Hu & Yu 2008).

In the context of the project ‘BEF (Biodiversity and Ecosystem Functioning) China’, 27 study plots of 30 × 30 m were established in the nature reserve (H. Bruelheide, M. Böhnke, S. Both, T. Fang, T. Assmann, M. Baruffol *et al.*, unpubl. data). Plot locations were randomly chosen within strata of different plot age from suitable forest stands distributed across the whole nature reserve, limited by inaccessibility and steep topography (areas with an inclination >55° were excluded) of parts of the reserve. In total there were nine replicates of each young (about 10–20 years old), middle-aged (about 40–50 years old) and old (>70 years old) forest stands, differing in species richness of trees and shrubs. Within each of the three successional stages, species richness of woody plants similarly varied between plots from a minimum of 25–30 to a maximum of 55–68 species. The scale of foraging of insect herbivores might vary between species and thus also the scale of perception of plant diversity. To account for this, we checked diversity patterns and their relationship with herbivory levels also for smaller subsamples of trees within the study plots. As a result of their status as a national nature reserve, the forest stands have not been managed over the last decades and thus have been subject to low anthropogenic influence.

### Study species and herbivory assessment

Ten evergreen tree and shrub species were selected to study folivory on saplings in relation to tree species diversity and stand characteristics: *Ardisia crenata* Sims, *Camellia fraterna* Hance, *Castanopsis eyrei* (Champ. ex Benth.) Tutch., *Cyclobalanopsis glauca* (Thunb.) Oerst., *Eurya muricata* Dunn, *Lithocarpus glaber* (Thunb.) Nakai, *Loropetalum chinense* (R. Br.) Oliv., *Machilus thunbergii* Sieb. et Zucc., *Neolitsea aurata* (Hayata) Koidz. and *Schima superba* Gardn. et Champ. These 10 species on average accounted for 40% of all individuals and 45% of the total biomass (as approximated by their local relative basal area) in the tree and shrub layers of the study plots. In each plot, a maximum of 10 saplings of each species (with a height between 20 and 100 cm, which was recorded for each sapling as a covariate for the statistical analysis) were randomly sampled by crossing the whole plot along parallel transects. All species were present and sampled in most of the plots and missing values for single species in single plots were accounted for in the statistical analysis. The degree of foliar damage by insects, defined as the combined removal of photosynthetic tissue by leaf-chewing, mining and galling (and, if visible, sucking) insects, was assessed for all leaves of the saplings to estimate overall damage levels for each individual. Most folivory damage could clearly be attributed to feeding patterns caused by mainly herbivorous lepidopterans and several beetle families observed during the assessment. Only senescent leaves or leaves heavily damaged by fungi were excluded from the assessment.

Sampling was conducted once on each plot at the end of the rainy season in June–July 2008, recording standing levels of insect herbivory (Blundell & Peart 1998). Although these are not necessarily representative of total annual herbivory, sampling at the end of the rainy season represents the degree of damage during one of the most important parts of the growing season, when water availability is best for plant growth, and when herbivory might thus have the greatest impact (Coley & Barone 1996; Hawkes & Sullivan 2001). Insect herbivore damage was estimated using percentage classes of herbivore damage (White & Whitham 2000; Scherber *et al.* 2006b; Vehviläinen, Koricheva & Ruohomaki 2007; Sobek *et al.* 2009). Each leaf was assigned to one of six percentage classes of tissue removal (0%, <1%, 1–5%, >5–15%, >15–35% and >35%). The six classes were defined beforehand and appropriateness of the estimates was checked by analysing samples of randomly collected leaves. The latter were digitally scanned and the degree of herbivory was determined using Adobe Photoshop CS3 to calculate pixel ratios of removed to estimated total tissue of each leaf (cf. Unsicker *et al.* 2006). Herbivore damage was assessed by one person only (A.S.) to prevent variability in estimation accuracy. In the statistical analyses, we used mean percentage of herbivory from the sampled and scanned leaves for each percentage class (0%, 0.5%, 3%, 9%, 23% and 55%).

### Predictors of herbivore damage

To test whether tree species diversity or other environmental parameters are able to explain differences in herbivory between the 27 forest stands, we used variables representing important plot characteristics, recorded during the 2008 growing season. Species richness of woody plants was based on the complete inventory of all tree and shrub individuals >1 m height in the plots. Diameter at breast height (d.b.h.) was recorded for all trees >10 cm d.b.h. in the whole plot and for all individuals >3 cm d.b.h. in a central plot of 10 × 10 m. From these data, we calculated sums of species-specific basal area as well as the total basal area of all trees and shrubs per plot as approximations of plant biomass. To test whether the biomass of the respective species (i.e. the concentration of this specific food resource) affects overall herbivory, we used the local relative basal area of conspecifics in the tree and shrub layers of each plot (which was strongly correlated with the absolute basal area of the target species in the study plots: *r* = 0.94, *P* < 0.001), henceforth referred to as ‘dominance’ for the sake of simplicity. Variables representing structural and abiotic plot conditions were altitude, aspect (divided into linear north–south and east–west gradients), canopy and herb cover, stand age, tree density and total basal area per plot (Table 1).

**Table 1:** Component loadings and eigenvalues of principal components (PC) selected from PCA reduction analysis on environmental variables (most influential variables in bold)

Variables	PC1	PC2	PC3
Stand age	**0.88**	−0.24	−0.11
Total basal area	**0.82**	−0.15	−0.07
Tree density	−**0.72**	**0.56**	−0.14
Canopy cover	−**0.65**	0.23	0.24
Herb cover	−0.47	−**0.70**	−0.27
Altitude	0.45	**0.57**	−**0.51**
Aspect (east–west)	0.47	**0.50**	−0.06
Aspect (north–south)	0.41	0.14	**0.81**
Cumulative proportion explained (%)	40.1	59.1	72.7
Eigenvalue	3.21	1.52	1.10

### Statistical analysis

Analyses were performed using R 2.7.1 (R Development Core Team 2008). Percentage data of insect herbivore damage were arcsine-square-root-transformed and dominance of target species was log_10_-transformed to account for non-normal or heteroscedastic error terms in the analyses. For our analyses, we used mean herbivore damage per individual nested within species to account for non-independent measurements (see next). We checked for significant nonlinear relationships between herbivory and the predictors by analysing second-order polynomials of the predictors.

Prior to the analysis of herbivory patterns, we conducted a dimension reduction in the set of explanatory variables related to stand structural and abiotic conditions (including stand age) by principal components analysis (PCA), as we were primarily interested in the main effects of a combined set of abiotic variables. Variable reduction by PCA allows extraction of a set of uncorrelated principal components (PC) which represent a large fraction of the variability of the original variables in reduced dimensionality (Legendre & Legendre 1998; Quinn & Keough 2002). The analyses were conducted on the standardized values of the variables using a correlation matrix. All PCs with eigenvalues higher than the mean were selected for further analyses. Structural and abiotic variables were reduced to a condensed set of three PCs. PC1 primarily represented stand age as well as age-dependent aspects of stand structure and biomass, together with age-related effects of other abiotic conditions (Table 1). PC2 and PC3 summed up further effects of abiotic characteristics (altitude, herb cover and aspect) related to growing conditions (such as temperature and light availability) independent of stand age. The three PCs together explained 73% of the variation in the structural and abiotic variables. Results from the analyses using the reduced set of PCs were compared with results using all eight PCs in a backward elimination procedure and to results of partial least-squares regression to verify that all relevant information for the relationships between the stand structural and abiotic parameters and herbivore damage was included in the reduced set of PCs (see Appendix S1 in Supporting Information for details on these additional methods and results). None of the omitted PCs significantly contributed to the herbivory pattern, and comparing our results with the alternative analysis using partial least-squares regression for dimension reduction did not yield deviating results (Appendix S1).

Different insect herbivores might have different scales of perception of plant diversity. To assess whether diversity patterns and the species richness–herbivory relationship are consistent across different spatial scales at a subplot level, we analysed correlations between species richness of woody plants at the plot level and rarefied richness (for random draws of 20, 50, 100 and 150 plant individuals, respectively), as well as correlations between herbivory levels and the different richness measures. Rarefied values were calculated with the vegan package in R (Oksanen *et al.* 2008).

Herbivory patterns were analysed using linear mixed-effects models (Pinheiro & Bates 2000) in an information-theoretic approach (Burnham & Anderson 2004) with the package lme4 in R (Bates & Meaechler 2009). A maximal model, with all variables and interactions of interest, was fitted including stand age and structure (PC1 from the above PCA), abiotic plot conditions (PCs 2 and 3), species richness of trees and shrubs and dominance as fixed effects. We also included sapling height and the total number of leaves of each sapling to take into account sapling apparency or dilution effects on herbivory levels owing to differences in size or leaf number. As we were interested in the potential influence of stand age on richness or dominance effects, we also included the interactions between stand age and richness/dominance. Species identity, with individuals nested within species, and plot were considered as crossed random effects, taking into account the hierarchical structure of the data (Pinheiro & Bates 2000). We also tested for interactions between species identity and species richness of woody plants, comparing model fit with this interaction included to model fit without this interaction in the random-effects structure with a likelihood ratio test (Pinheiro & Bates 2000). The following three hypotheses were considered to explain differences in herbivore damage across plots: (a) both plot characteristics and richness/dominance, (b) plot characteristics only or (c) richness and/or dominance alone are important predictors of herbivory. As the number of predictors was small and any combination of the fixed variables might influence the degree of insect herbivory, we used an ‘all-subsets’ approach with information-theoretic selection criteria (Quinn & Keough 2002; Burnham & Anderson 2004), including the age ×richness/dominance interactions for those cases where both stand age and richness/dominance were present. Model fit was assessed and models were ranked based on Akaike’s information criterion (AICc for small sample sizes; Burnham & Anderson 2004). For each of the three hypotheses, we selected the three best-fit models, that is, those with lowest AICc values, and compared their performance calculating ΔAICc as the difference in AICc between the candidate and the best-fit model. Models with ΔAICc ≤ 2 are considered to be equally likely, whereas larger values indicate a lack of fit and lower explanatory power relative to the best model (Burnham & Anderson 2004). In case of differences ≤2, the model with the smaller number of predictors was preferred. The package language R with the function pvals.fnc for mixed models was used to assess the significance of the model parameters (Baayen 2009), with *P*-values based on Markov chain Monte Carlo sampling (Baayen, Davidson & Bates 2008). Model residuals were checked for modelling assumptions of normality and homogeneity of variances. Although mixed models are a powerful tool to deal with missing values (Pinheiro & Bates 2000) and species-specific effects can be tested with interaction terms, we additionally checked for a potential bias introduced by differential abundance of sapling species in the samples along the diversity gradient. The data were reanalysed after excluding *C. glauca*, the only species showing higher relative abundance in species-rich compared with species-poor plots.

## Results

**Fig. 1:**
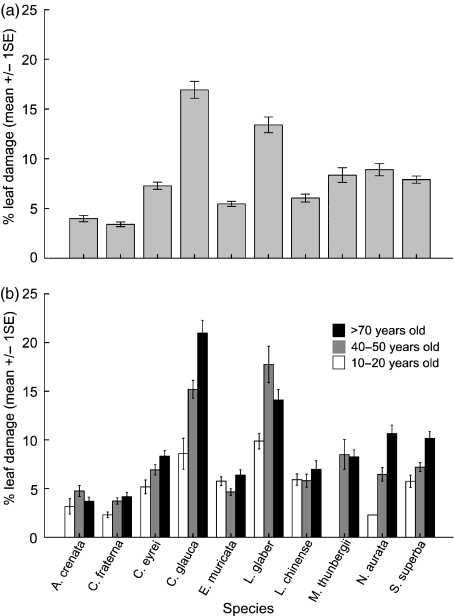
Mean percentage (±1 SE) of leaf consumption for saplings of the 10 study species (*Ardisia crenata, Camellia fraterna, Castanopsis eyrei, Cyclobalanopsis glauca, Eurya muricata, Lithocarpus glaber, Loropetalum chinense, Machilus thunbergii, Neolitsea aurata* and *Schima superba*): (a) mean values per plot; (b) mean values per age class.

In total, 58 297 leaves from 1284 saplings of the 10 study species were sampled. Mean herbivory of saplings differed between species, with highest damage levels (13–17%) in *C. glauca* and *L. glaber* and lowest levels (3–4%) in *A. crenata* and *C. fraterna* (Fig. 1a). Mean overall percentage of herbivory for the 10 species was 8%. Herbivory generally increased from the young to the older plots (Fig. 1b). Total species richness of the plots was strongly correlated with rarefied richness at all levels (Pearson correlations: *r*= 0.72/0.80/0.86/0.88 for correlations with mean richness of 20/50/100/150 plant individuals; *P* < 0.01 in all cases). Similarly, mean herbivory levels were strongly related to species richness of woody plants at all levels (*r* = 0.52/0.56/0.59/0.60; *P* < 0.01 for mean richness of 20/50/100/150 plant individuals and *r* = 0.48; *P* < 0.05 for species richness at the plot level), indicating that diversity and herbivory relationships at smaller scales are well represented by our analysis at the plot level.

Mixed-effects modelling showed a substantially better fit of models including both plot characteristics and species richness rather than only one of these (Table 2). The best-fit models contained both stand age and structure (PC1, which primarily reflects stand age-related differences in biotic and abiotic conditions) and species richness as predictors, and the model with only these two variables had the lowest AICc. Models with an ΔAICc ≤ 2 additionally included one more predictor, either dominance or the PC1–richness interaction. However, the estimated effects of these variables were not significantly different from zero (Table 2). The same was true for effects of abiotic plot conditions other than stand age-related effects and of dominance or sapling height in the models considering only plot characteristics or only richness and dominance. Stand age and structure (PC1) and species richness were the most important parameters in these models as well. There was no effect of potential interactions between species identity and species richness. Including this interaction in the random-effects structure did not significantly improve model fit (likelihood ratio test with χ² = 4.7; d.f. = 2; *P* = 0.1). Relative abundance of most sapling species was constant over the samples of the 27 plots. Only the proportion of *C. glauca* saplings significantly increased with increasing species richness of the plots (*F*_1,25_ =12.1, *P* = 0.001), whereas the proportion of *L. chinense* decreased (*F*_1,25_=5.2; *P* = 0.031). As *C. glauca* featured highest damage levels of all species studied (whereas *L. chinense* showed medium herbivore damage; Fig. 1a), the data were reanalysed after excluding *C. glauca* saplings. However, mixed-model results did not differ from those of the overall analysis (see Appendix S2). There was also no significant effect of sapling proportions on herbivory levels for any of the species (not shown).

**Table 2:** Results from linear mixed-effects modelling. For each predictor set (a–c), the three best-fit models (lowest AICc) are shown, with regression estimates (±SE) for the predictors included.^a^ΔAICc (Akaike information criterion) is the difference in AICc values between the candidate and the overall best-fit (in bold) model. Estimated effects of predictors in italics are not significantly different from zero (based on Markov chain Monte Carlo sampling)

Models	AICc	ΔAICc
(a) Plot characteristics and species richness		
**0.0116 (±0.0031) PC1 + 0.0012 (±0.0005) richness**	−**2555.8**	**0**
−*0.0028 (±0.0144) PC1 + 0.0011 (±0.0006) richness + 0.0004 (±0.0004) PC1 × richness*	−2555.0	0.8
0.0115 (±0.0030) PC1 + 0.0012 (±0.0005) richness*−0.0030 (±0.0036) dominance*	−2554.3	1.5
(b) Only plot characteristics		
0.0126 (±0.0032) PC1	−2552.6	3.2
0.0126 (±0.0032) PC1*−0.0022 (±0.0054) PC3*	−2550.7	5.1
0.0126 (±0.0032) PC1*−0.0043 (±0.0119) sapling height*	−2550.6	5.2
(c) Only species richness and dominance		
0.0015 (±0.0006) richness	−2545.8	10.0
0.0015 (±0.0006) richness−*0.0037 (±0.0036) dominance*	−2544.8	11.0
0.0015 (±0.0006) richness*−0.0041 (±0.0119) sapling height*	−2544.2	11.6

a PC1, principal component 1 from PCA dimension reduction (Table 1), primarily reflecting stand age-related differences in biotic and abiotic conditions; PC3, principal component 3 (see Table 1); richness, species richness of trees and shrubs; PC1*×*richness, interaction between stand age/structure and species richness.

**Fig. 2:**
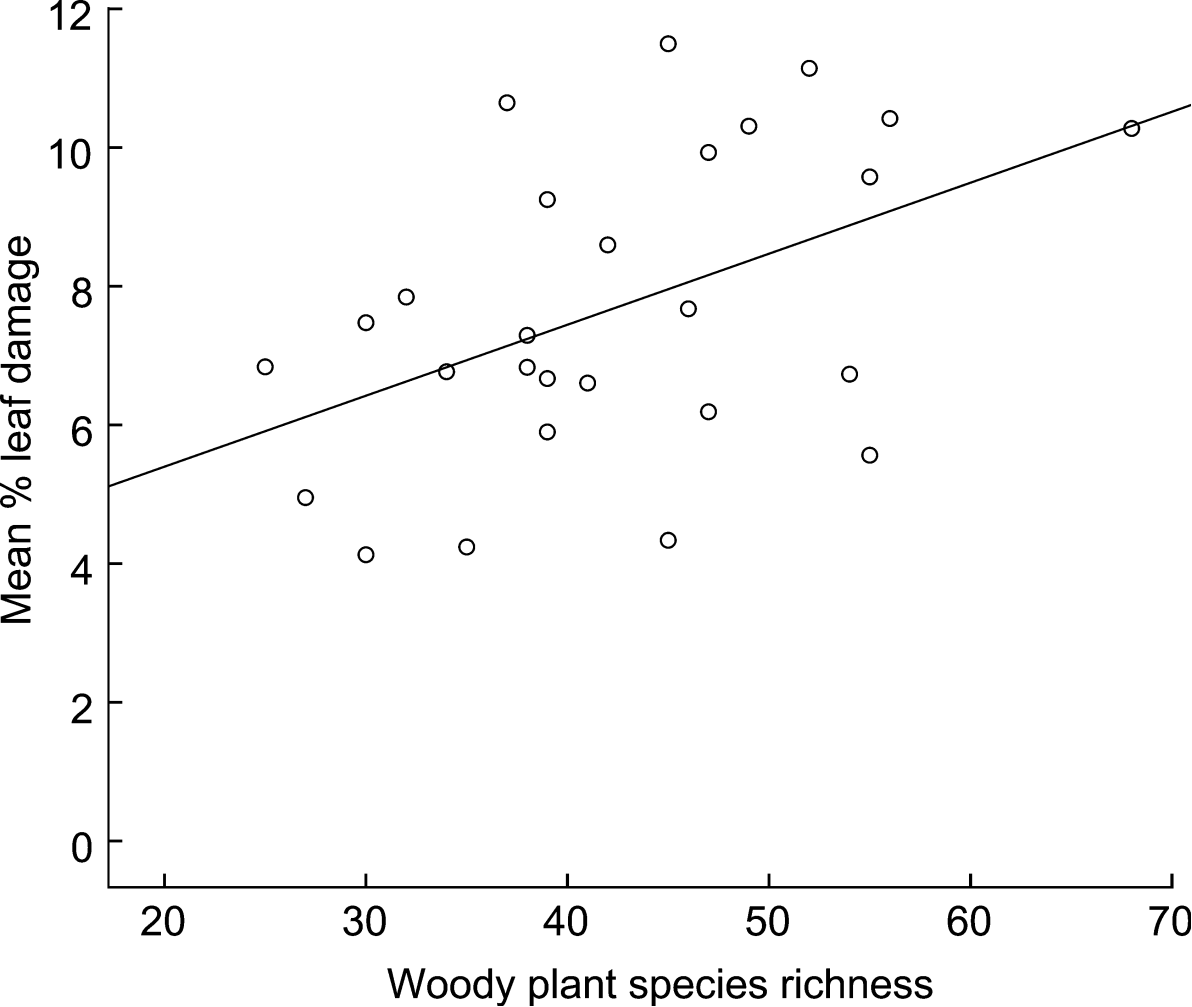
Mean percentage of leaf damage per plot owing to insect herbivory in relation to species richness of trees and shrubs across a diversity gradient of 27 study plots in subtropical China (β = 0.001, *P* = 0.025).

**Fig. 3:**
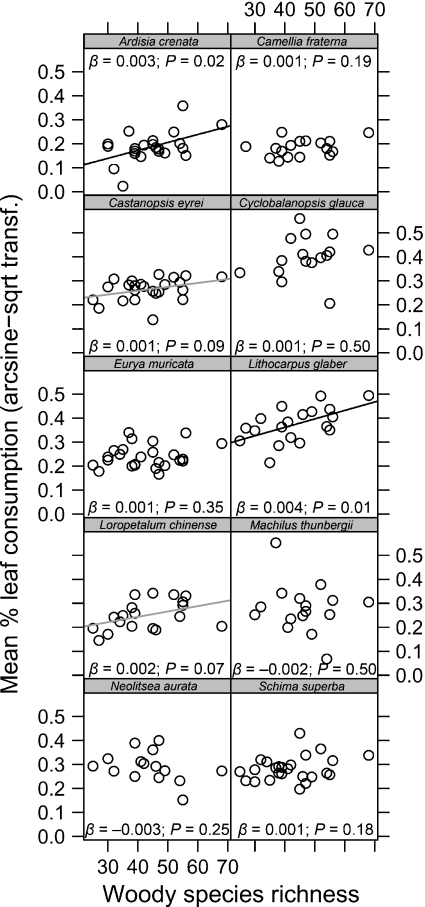
Relationship between herbivore damage of the single study species (arcsine-square-root-transformed) and species richness of woody plants across the diversity gradient of 27 study plots in subtropical China. Regression slopes (β; with their probabilities *P*) from single regressions show sign and magnitude of the relationships; regression lines indicate significant (black) and close to significant (grey) relationships.

The best mixed model in our analysis, with species richness and stand age and structure (PC1) as fixed effects, accounted for 41% of the variation in the herbivory data. Species identity accounted for most of the variation in the random-effects structure (38.7% as compared with the plot effect of 3.8%). This strong intercept effect underlines the large differences in species-specific susceptibility to insect herbivory (cf. Fig. 1a). The degree of herbivory over all species was positively related to both stand age and structure and woody plant species richness. As can be inferred from this model, however, stand age and structure had a stronger impact on herbivore damage than species richness. This is also shown by the substantially smaller AICc value for the model including only stand age and structure compared with the model including only richness (Table 2). With respect to plant species richness, our model predicts an increase in herbivory of about 1% with an increase in richness by 10 tree and shrub species (Table 2 and Fig. 2). Across the richness gradient of the 27 study plots, this amounts to an overall increase in mean herbivore damage of about 5% from the least to the most species-rich plots, which is a doubling of mean herbivory over all species from about 5% to 10% (Fig. 2). Although mixed-model results indicate a general and significant overall increase in herbivore damage, single regressions for the study species show that this pattern was most pronounced for *L. glaber* and *A. crenata* and also for *C. eyrei* and *L. chinense* (Fig. 3). Even though not significant in the single species analyses, most other species also showed a tendency towards increasing damage with higher stand diversity, with regression estimates of β = 0.001 close to the overall mixed-model estimate. Only patterns in *M. thunbergii* and *N. aurata* slightly deviated (Fig. 3). However, this did not have an effect on the overall pattern, as is also shown by the better fit of the mixed model excluding separate parameters for each species through a species–richness interaction. Significance levels in the single regressions must be interpreted with care, as they are subject to multiple testing. However, although with α=0.05 we would expect one species out of 20 to show a significant effect by chance, our analysis finds two significant relationships in 10 species, which additionally have the same direction. The mixed-model results further confirm the general positive relationship between plant diversity and herbivory.

Measures of rarity could not explain the species-specific susceptibility to herbivory: the mean rate of herbivore damage of the 10 species analysed was not related to their general dominance in the studied forest ecosystem (i.e. the basal area each species accounted for across all 27 study plots; Pearson correlation *r* = −0.03, *P* = 0.93).

## Discussion

Although observational studies have to take a range of potentially confounding factors into account, they have the advantage of providing near-natural conditions and fully established communities of animals and plants with a multitude of interactions that might not have developed in the same way in artificial experiments (Scherber *et al.* 2006b; Unsicker *et al.* 2006; Leuschner, Jungkunst & Fleck 2009). This is especially important for the study of ecosystems dominated by long-lived individuals, such as forests, where successional processes can strongly influence the outcome of analyses (Leuschner, Jungkunst & Fleck 2009). With a paucity of long-term experimental setups studying the relationship between biodiversity and ecosystem functions in forests, observational studies provide essential insights into the role of biodiversity in influencing processes and interactions in these ecosystems (Scherer-Lorenzen, Körner & Schulze 2005; Leuschner, Jungkunst & Fleck 2009).

### Effects of plant species richness on herbivory

Our study of foliar damage on saplings in subtropical forests shows that tree species diversity can be important in influencing insect herbivory even in an extremely species-rich forest ecosystem. Interestingly, our results indicate an altogether positive relationship between plant diversity and herbivory in these forest stands. This is in contrast to many studies reporting a decrease in herbivory with increasing plant diversity both in forests and in other systems (Andow 1991; Hambäck, Agren & Ericson 2000; Massey *et al.* 2006; Unsicker *et al.* 2006; Jactel & Brockerhoff 2007; Sobek *et al.* 2009). However, these studies usually considered gradients of species richness in plant communities much less diverse than those of our study, the latter of which feature a relatively high species richness already in the least diverse plots. Hypotheses to explain the pattern reported in the aforementioned studies comprise those on resource–concentration and on predator effects formulated by Root (1973). In many of the studied systems, specialized herbivores are considered to cause the largest amount of damage to plants. The resource–concentration hypothesis, originating from agroecosystem studies, but often also considered relevant for other systems, predicts lower specialist herbivore loads (and, as generally assumed, lower resulting plant damage) in more diverse plant communities. Specialists might have difficulties locating their hosts in more diverse stands owing to reduced host plant abundance and distracting effects (e.g. optical, olfactory) of non-host plants. Herbivore communities of the highly phytodiverse forests occurring in the tropics and subtropics are also typically considered to be dominated by specialized, oligophagous species with a much stronger impact on damage levels than polyphagous species (Erwin 1982; Barone 1998, 2000; Dyer *et al.* 2007). Considering this theory, we might thus have expected lower herbivore damage in the more diverse stands of our study.

However, the fact that we found the opposite pattern is consistent with a range of recent studies (Mulder *et al.* 1999; Prieur-Richard *et al.* 2002; Scherber *et al.* 2006b; Vehviläinen, Koricheva & Ruohomaki 2007). In these studies (mainly of grassland systems), this positive effect was also repeatedly found over higher gradients of plant diversity than usually considered (where diversity effects often depend on the identity of the plants added to a system; cf. Unsicker *et al.* 2006). One reason for this pattern could be a higher impact of generalist, polyphagous insects and a comparatively lower influence of specialist herbivores in these systems (Basset 1999; White & Whitham 2000; Jactel & Brockerhoff 2007). Polyphagous herbivores can profit from and are able to cause greater damage in more diverse stands, which provide a greater variety of resources for these taxa (Pfisterer, Diemer & Schmid 2003; Joshi *et al.* 2004; Jactel & Brockerhoff 2007; Unsicker *et al.* 2008). White & Whitham (2000) suggested the use of the term ‘associational susceptibility’ for those cases where plant species experience an increase in herbivore damage from a spill over (which might occur for several reasons such as resource depletion or preference shifts) of generalist herbivores feeding on neighbouring plant species. Associational susceptibility depends on the host preferences of the generalist herbivore and the palatability of the respective plant species (White & Whitham 2000). Higher plant diversity increases the probability of suitable plant species associations for generalist herbivores and thus the probability of higher damage in systems dominated by generalist herbivores. A possible explanation for our findings could thus be a much stronger impact of generalist herbivores on overall herbivore damage in our subtropical forest than often hypothesized for such highly diverse systems. Such strong effects of generalists on herbivory or a less narrow specialization of the herbivore community than traditionally assumed has also been found to occur in similarly species-rich tropical forests (Basset 1999; Novotny *et al.* 2002; Novotny & Basset 2005). Even though we lack systematic data on feeding preferences of herbivorous insects for our study region, observations during our census show that single species which feed on a broad range of tree species can cause substantial leaf damage. For instance, adults of the curculionid *Heterapoderus sulcicollis* (Jekel 1860) were observed feeding on at least eight tree species of different genera and families. In comparison, related weevils from temperate regions are more restricted to single or few tree species (cf. Kippenberg 1981). As we recorded total leaf damage, we cannot directly differentiate between effects of different functional herbivore groups. However, the largest part of the damage was caused by leaf-chewing lepidopterous larvae and beetles (see ‘Materials and methods’), taxa which are often considered to comprise many generalist feeders also in tropical forests (Novotny & Basset 2005). Future work in the forest stands and on the newly established tree plantations of the BEF China project will further clarify dietary preferences of dominant insect herbivores and specific effects of different functional groups.

Supporting evidence for our findings also comes from our consideration of potential effects of target species dominance. The Janzen–Connell hypothesis (Janzen 1970; Connell 1971) predicts less strong effects of specialist herbivores on growth and mortality of saplings when young trees grow at a greater distance from adult trees of the same species or at lower densities of neighbouring conspecifics. A recent meta-analysis by Hyatt *et al.* (2003) found no general support for intraspecific distance dependence of herbivory on saplings, which was present in only some of the species studied so far. Distance and density effects probably depend on the herbivore community (i.e. the dominance of specialists or generalists) associated with each species (Barone 2000). The lack of a positive effect of the dominance of the target species in the tree and shrub layers in our study thus also suggests that polyphagous insect herbivores indeed play an important role in determining herbivory levels in highly diverse subtropical forests. At least in the sum of herbivory effects, which as a whole influence fitness and survival of the saplings, resource–concentration effects related to the hypotheses of Root or Janzen and Connell do not seem to have a strong impact on the overall damage levels of the 10 species studied in our subtropical forest system. Likewise, effects predicted from the natural enemies’ hypothesis do not appear to significantly influence overall herbivory levels observed in our study system. This hypothesis suggests that greater diversity and abundance of predators with increasing plant diversity causes a reduction of herbivore loads and damage in more diverse plant communities, because of a broader spectrum and a temporally more stable availability of prey as well as a greater variety of niches (Root 1973). Although support for this hypothesis has been reported from several studies of less diverse systems (e.g. Sobek *et al.* 2009), the effect of predators might depend on the system studied (Vehviläinen, Koricheva & Ruohomaki 2007). We presently lack information on predator diversity in our plots and further research is needed to evaluate predator–herbivore relationships in our study system.

### Effects on single species

The strength of positive relationships between plant diversity and herbivore damage varied to some extent between species. This might be the result of differences in the palatability of the species and the feeding preferences of dominant generalist herbivores, such that the study species show different degrees of associational susceptibility (White & Whitham 2000). Our study treated sapling species as random effects, so that the general positive trend of herbivore damage with tree species richness would be expected for a randomly drawn species of the dominant tree species pool (cf. Pinheiro & Bates 2000). The mixed model shows that differences between species in their response to plant diversity are consistent with the assumption of an increase of herbivore damage with diversity, independent of species identity. As proportions of the heavily damaged *C. glauca* increased in the samples of the more diverse stands, this species might have potentially affected the overall results. However, the mixed-model analysis takes into account potential species-specific effects, and results from the additional reanalysis of the data after excluding *C. glauca* did not differ from the overall analysis, showing that the patterns found were not affected by distribution characteristics of, for example, particularly herbivore-prone species.

As specified in ‘ Materials and methods’, these 10 species belong to the most common plant species in our subtropical forest ecosystem. Theories on the origin of the extraordinary species richness of subtropical and tropical forests suggest that herbivorous insects might contribute to high plant diversity by differentially affecting common and rare plant species (Janzen 1970; Connell 1971; Givnish 1999). The newly established tree plantations of the BEF China project might help to further investigate this issue, as we are unable to fully test these assumptions with our current data (rare species are difficult to sample in statistically sufficient numbers in our forest stands). For our 10 study species, we did not find indications of decreasing susceptibility to herbivory with decreasing commonness of the species.

Whether the species richness effect suggested by our results can significantly affect, either by itself or in an interaction with other factors, the fitness or survival of the saplings analysed depends on the capacities of the single species to compensate leaf damage and requires further testing. Species already experiencing high levels of herbivory (e.g. *L. glaber*) might be more affected by an increase in herbivory across the diversity gradient than species with generally low damage levels (e.g. *C. fraterna*). However, effects on plant fitness have already been found at even lower damage levels than those reported in our study (Scherber *et al.* 2006a). Altogether, as our study species accounted on average for 40% of all plant individuals in the tree and shrub layers, the overall positive effect of plant species richness on herbivory applies to a substantial part of the plant communities of the studied subtropical forest system.

### Effects of stand age and abiotic conditions

Models considering both species richness and plot conditions were the best fit in our statistical analysis, and stand age and structure played an important role in these models. Herbivore damage increased with stand age, which was the main factor with the highest loading on the first PC in the dimension reduction of structural/abiotic variables. At the same time, tree density and canopy cover were negatively related and total basal area of trees and shrubs were positively related to stand age. Moreover, several other abiotic variables covaried with stand age. It is well known that abiotic conditions as well as the composition of and interactions within the biotic community, and thus their potential impact on herbivory, change with the successional development of an ecosystem (Poorter *et al.* 2004; Vilà*et al.* 2005; Vehviläinen, Koricheva & Ruohomaki 2007; Leuschner, Jungkunst & Fleck 2009). However, even after accounting for these strong effects, we did find significant effects of plant diversity on herbivory. Beyond age-related differences, variables related to important abiotic conditions which might, directly or indirectly, influence herbivory (e.g. light and temperature conditions; Coley & Barone 1996; Howlett & Davidson 2001) did not have additional explanatory power. This suggests that the gradient in abiotic conditions across the 27 study plots may be too small to have a severe impact on either leaf quality or herbivore communities of our study species beyond stand age-related effects (see also Howlett & Davidson 2001; Eichhorn, Compton & Hartley 2006). It further indicates that, despite the observational character of our study, the positive effects of stand diversity on herbivory are unlikely to be artificially caused by underlying and covarying abiotic conditions of the plots.

## Conclusions

Our study documents a positive relationship between the degree of insect herbivory and plant diversity in a highly diverse subtropical forest system for a substantial part of the whole plant community, even after accounting for stand age and environmental variability. Our findings of increasing herbivory with an increase in plant species richness suggest that effects expected from classical hypotheses on plant diversity–herbivory relationships do not seem to play a major role in overall herbivory patterns of the species analysed in our study system. One possible mechanism explaining the contrasting results of other herbivory studies could be differences in the degree of host specificity of dominant species in the herbivore community, indicating a higher impact of generalist herbivores in our study than usually assumed for such phytodiverse forests. Additionally, our study comprises levels of plant diversity beyond the scope of most forest plantation experiments or previous observational studies. Of course our results are limited to above-ground herbivory of young understorey trees, but as the performance of saplings determines diversity of the forest stands in the long run, they are essential for a general understanding of plant–herbivore interactions in forests. Large-scale experimental studies focusing on similar systems, such as the newly established tree plantations of the BEF China project (H. Bruelheide, M. Böhnke, S. Both, T. Fang, T. Assmann, M. Baruffol *et al.*, unpubl. data), will help to establish the causative mechanisms underlying these patterns.

## Supporting Information

**Appendix S1.** Details onmethods and results of PCAvalidation.

**Appendix S2.** Mixed-model results excluding C. glauca.

As a service to our authors and readers, this journal provides supporting information supplied by the authors. Such materials may be re-organized for online delivery, but are not copy-edited or typeset. Technical support issues arising from supporting information (other than missing files) should be addressed to the authors.
