# Supplementary material for: Tree diversity promotes insect herbivory in subtropical forests of south-east China
Source: J Ecol. 2010 Jul;98(4):917–26. doi: 10.1111/j.1365-2745.2010.01659.x (PMC2936109; doi:10.1111/j.1365-2745.2010.01659.x)
Supplement: Supplementary file 1 [file jec0098-0917-SD1.pdf]

SUPPORTING INFORMATION

Schuldt et al. – Tree diversity promotes insect herbivory in subtropical forests of south-east China

Appendix S1 Details on methods and results of PCA validation

The parameters most strongly correlated with herbivory were stand age, total basal area, tree density and canopy cover (Table S1), all of which loaded most strongly on PC1, the only PC retained in the all-subsets analysis. Herbivory increased with stand age and total basal area, while it decreased with tree density and canopy cover.

Table S1. Component loadings and eigenvalues of all principal components (PC) from PCA reduction analysis on environmental variables as well as Pearson correlations of the single parameters and of each PC with mean herbivory per plot

Variable	*Correlation with leaf damage*	*PC 1*	*PC 2*	*PC 3*	*PC 4*	*PC 5*	*PC 6*	*PC 7*	*PC 8*
Stand age	r=0.66; p<0.001	0.88	-0.24	-0.11	0.26	-0.09	-0.10	0.12	0.24
Total basal area	r=0.55; p=0.003	0.82	-0.15	-0.07	0.21	0.42	0.08	0.21	-0.16
Tree density	r=-0.55; p=0.003	-0.72	0.56	-0.14	0.07	-0.17	0.00	0.36	0.01
Canopy cover	r=-0.54; p=0.003	-0.65	0.23	0.24	0.38	0.53	0.11	-0.08	0.12
Herb cover	r=-0.13; p=0.504	-0.47	-0.70	-0.27	-0.20	0.03	0.41	0.08	0.06
Altitude	r=0.03; p=0.866	0.45	0.57	-0.51	0.26	-0.18	0.31	-0.15	-0.01
Aspect (East-West)	r=0.22; p=0.254	0.47	0.50	-0.06	-0.65	0.32	0.05	0.02	0.09
Aspect (North-South)	r=0.25; p=0.206	0.41	0.14	0.81	0.00	-0.24	0.30	0.05	0.00
Cumulative proportion explained (%)		*40.1*	*59.1*	*72.7*	*82.5*	*91.2*	*96.0*	*98.8*	*100*
Eigenvalue		*3.21*	*1.52*	*1.09*	*0.78*	*0.69*	*0.38*	*0.22*	*0.11*
Corrleation with leaf damage (Pearson's r)		0.64	-0.27	0.04	-0.10	-0.03	-0.11	0.20	0.09
Probability		<0.001	0.165	0.841	0.629	0.871	0.580	0.315	0.645

Retaining Principal Components (PCs) from PCA dimension reduction with eigenvalues higher than the mean is a standard approach to select those PCs with highest information content for further analysis (Quinn & Keough 2002). However, additional PCs might theoretically be relevant in a regression on a response variable, as PCs are primarily optimised to account for the variation in the predictor and not directly in the response variable. We thus tested the robustness of our method and the results using two different approaches.
First of all, we tested whether inclusion of any of the PCs with eigenvalues <1 and not chosen in our all-subsets analysis significantly contributed to explaining the variance in the herbivory data. Starting with the maximal model of our all-subsets approach, as defined in the Materials and Methods section, but including all PCs instead of only the first three, we conducted a

mixed model analysis with backward elimination. Unlike with the original variables, including all eight PCs is possible as the components are orthogonal and thus there is no collinearity and instability in the estimates of the regression coefficients (Quinn & Keough 2002). Using backward elimination was preferred over an all-subsets approach as the number of predictors was much higher than in the all-subsets analysis using only three PCs. Insignificant variables were removed from the maximal model one by one, with each step checked by a likelihood-ratio test, until a minimal adequate model was obtained where further deletion of parameters would have lead to significant changes in deviance (Scherber *et al.* 2006; Crawley 2007). The minimal model obtained was identical to the best-fit model in the all-subsets analysis using only the PCs with eigenvalues higher than the mean (cf. Table 2), with only PC1 and species richness retained (Table S2). All other PCs did not significantly increase model fit and were successively removed, showing that our analysis using only the first three PCs includes all relevant information on stand structural and abiotic aspects for the analysis of herbivory patterns.

Table S2. Results for the minimal adequate model of mixed effects modelling with backward elimination*

Fixed effects	*Estimate*	*Std. Error*	*t value*	p_{MCMC}
Intercept	0.2108	0.0329	6.40	0.001
Woody plant species richness	0.0012	0.0005	2.35	0.0198
PC1	0.0116	0.0031	3.79	0.008

*starting with a maximal model including as fixed effects: PCs1-8 (cf. Table S1), species richness, dominance, sapling height and number of leaves, interactions of richness and dominance with PC1.

Additionally, we compared our results to a mixed model approach using components for stand structural and abiotic aspects derived from partial least squares (pls) regression. Pls regression yields components based on the direct relationship between the predictors and the response variable (Mevik & Wehrens 2007). However, it is not possible to take into account the hierarchical structure in our dataset (species and individuals nested in plots) with the pls regression procedure and it is not able to handle missing values intrinsically (Mevik & Wehrens 2007). In our case, pls regression is only possible on mean values of herbivory (averaged over all species) per plot and component loadings do not directly reflect effects on species-specific levels of herbivory (and might thus introduce a bias into the estimation of regression slopes). We thus performed a pls regression of stand structural and abiotic parameters (cf. Table 1) on mean herbivory values per plot, using the package pls for R (Wehrens & Mevik 2007). Components relevant for the explanation of the variation in herbivory were selected on the basis of the root mean squared error of prediction (RMSEP) (Mevik & Wehrens 2007). The pls separated our first PC axis into three orthogonal components, with total basal area loading strongest on PLS1, tree density loading strongest on

PLS2, and stand age loading strongest on PLS3 (Table S3). Given that PLS1 explains most variance in herbivory, followed by PLS3, one would come to the conclusion, that the effect of total basal area can be separated from stand age. However, since the pls was performed on mean values within plots, these results may also reflect a simple bias in species incidence within plots. The interpretation is further complicated by the contrasting results when including the PLS components into the mixed model together with species richness.

Table S3. Loadings of the main variables on the components (PLS1-3) selected from partial least squares (pls) regression

Variable	*PLS1*	*PLS2*	*PLS3*
Stand age			0.80
Total basal area	1.00		
Tree density		-0.95	0.16
Canopy cover			-0.53
Herb cover			-0.29
Altitude		-0.33	
Aspect (East-West)			
Aspect (North-South)			
Sum of squares loadings	1.00	1.01	1.02
Cumulative Variance Explained in the response variable	30.24	37.04	53.54

We used the three components in an all-subsets mixed model approach, with the same model structure as in the all-subsets analysis using PCs (see Materials and Methods), but substituting the three pls components for the PCs. The best-fit model contained species richness and pls components PLS3 and PLS1 (Table S4). Component PLS3, with high loadings of primarily stand age and to a lesser degree canopy cover, had a much higher effect on herbivory than component PLS1, which reflected total basal area of the plots. Thus, the importance of PLS3 and PLS1 flipped when accounting for the nested structure of the data as well as for diversity. The interpretation of the two PLS axes is difficult, since they may include a bias of species incidence within plot (see above). Results are still very similar to the all-subsets approach using PCs (Table 2), with both approaches identifying species richness and the components related most strongly to stand age and total basal area as the most important variables.

Table S4. Results from linear mixed-effects modelling using data from partial least squares (pls) regression. For each predictor set (a-c), the three best-fit models (lowest AICc) are shown, with regression estimates (± standard error) for the predictors included*. ΔAICc is the difference in AICc-values between the candidate and the overall best-fit (in bold) model. Estimated effects of predictors in italics are not significantly different from zero (based on Markov chain Monte Carlo sampling)

Model	AICc	ΔAICc
a) Plot characteristics and species richness		
**0.0007 (±0.0004) PLS3+0.0011 (±0.0006) richness+0.000002 (±0.0000006) PLS1**	**-2554.8**	**0**
0.0007 (±0.0004) PLS3+0.0011 (±0.0007) richness+0.000002 (±0.0000006) PLS1-*0.0029 (±0.0036) dominance*	-2553.4	1.4
0.0007 (±0.0003) PLS3+0.0011 (±0.0005) richness+0.000002 (±0.0000005) PLS1-*0.0053 (±0.0122) sapling height*	-2553.0	1.8
b) Only plot characteristics		
0.0009 (±0.0003) PLS3+0.000002 (±0.0000005) PLS1	-2552.7	2.1
0.0009 (±0.0003) PLS3+0.000002 (±0.0000006) PLS1-*0.0042 (±0.0121) sapling height*	-2550.8	4.0
0.0009 (±0.0003) PLS3+0.000002 (±0.0000006) PLS1-*0.0000005 (±0.00002) PLS2*	-2550.7	4.1
c) Only species richness and dominance		
0.0015 (±0.0006) richness	-2545.8	9.0
0.0015 (±0.0007) richness-*0.0037 (±0.0036) dominance*	-2544.8	10.0
0.0015 (±0.0006) richness-*0.0041 (±0.0119) sapling height*	-2544.2	10.6

*PLS1, PLS2, PLS3: Components 1, 2 and 3 from pls regression (Table S3); richness: species richness of trees and shrubs; PLS3:richness: interaction between stand age/structure and species richness.

References

Crawley, M.J. (2007) *The R Book* Wiley, Chichester.

Mevik, B.H. & Wehrens, R. (2007) The pls Package: Principal Component and Partial Least Squares Regression in R. *Journal of Statistical Software*, **18**, 1-24.

Quinn, G.P. & Keough, M.J. (2002) *Experimental Design and Data Analysis for Biologists* University Press, Cambridge.

Scherber, C., Mwangi, P.N., Temperton, V.M., Roscher, C., Schumacher, J., Schmid, B., & Weisser, W.W. (2006) Effects of plant diversity on invertebrate herbivory in experimental grassland. *Oecologia*, **147**, 489-500.

Wehrens, R. & Mevik, B.H. (2007) pls: Partial Least Squares Regression (PLSR) and Principal Component Regression (PCR). R package version 2.1-0. http://mevik.net/work/software/pls.html.
