# Supplementary material for: Tree diversity promotes insect herbivory in subtropical forests of south-east China
Source: J Ecol. 2010 Jul;98(4):917–26. doi: 10.1111/j.1365-2745.2010.01659.x (PMC2936109; doi:10.1111/j.1365-2745.2010.01659.x)
Supplement: Supplementary file 2 [file jec0098-0917-SD2.pdf]

SUPPORTING INFORMATION

Schuldt et al. – Tree diversity promotes insect herbivory in subtropical forests of south-east China

Appendix S2 Mixed model results excluding *C. glauca*

Table S5. Results from linear mixed-effects modelling excluding data from *Cyclobalanopsis glauca* (Thunb.). For each predictor set (a-c), the three best-fit models (lowest AICc) are shown, with regression estimates (± standard error) for the predictors included*. ΔAICc is the difference in AICc-values between the candidate and the overall best-fit (in bold) model. Estimated effects of predictors in italics are not significantly different from zero (based on Markov chain Monte Carlo sampling)

Model	AICc	ΔAICc
a) Plot characteristics and species richness		
**0.0110 (±0.0030) PC1+0.0012 (±0.0005) richness**	**-2423.7**	**0**
-0.0031 (±0.0129) PC1+0.0010 (±0.0005) richness+0.0003 (±0.0003) PC1:richness	-2422.8	0.9
0.0110 (±0.0030) PC1+0.0012 (±0.0005) richness*-0.0117 (±0.0123) sapling height*	-2422.5	1.2
b) Only plot characteristics		
0.0118 (±0.0033) PC1	-2421.0	2.7
0.0118 (±0.0033) PC1*-0.0110 (±0.0123) sapling height*	-2419.6	4.1
0.0118 (±0.0033) PC1*-0.0038 (±0.0075) number of leaves*	-2419.1	4.6
c) Only species richness and dominance		
0.0015 (±0.0006) richness	-2415.6	8.1
0.0015 (±0.0006) richness*-0.0105 (±0.0123) sapling height*	-2414.1	9.6
0.0015 (±0.0006) richness*-0.0025 (±0.0038) dominance*	-2413.8	9.9

*PC1: Principal component 1 from PCA dimension reduction (Table 1), primarily reflecting stand age-related differences in biotic and abiotic conditions; PC3: Principal component 3 (see Table 1); richness: species richness of trees and shrubs; PC1:richness: interaction between stand age/structure and species richness
